# MICAN : a protein structure alignment algorithm that can handle Multiple-chains, Inverse alignments, C_*α*_ only models, Alternative alignments, and Non-sequential alignments

**DOI:** 10.1186/1471-2105-14-24

**Published:** 2013-01-18

**Authors:** Shintaro Minami, Kengo Sawada, George Chikenji

**Affiliations:** 1 Department of Computational Science and Engineering, Nagoya University, Nagoya 464-8603, Japan; 2 Department of Applied Physics, Nagoya University, Nagoya 464-8603, Japan

## Abstract

**Background:**

Protein pairs that have the same secondary structure packing arrangement but have different topologies have attracted much attention in terms of both evolution and physical chemistry of protein structures. Further investigation of such protein relationships would give us a hint as to how proteins can change their fold in the course of evolution, as well as a insight into physico-chemical properties of secondary structure packing. For this purpose, highly accurate sequence order independent structure comparison methods are needed.

**Results:**

We have developed a novel protein structure alignment algorithm, MICAN (a structure alignment algorithm that can handle Multiple-chain complexes, Inverse direction of secondary structures, C_*α*_ only models, Alternative alignments, and Non-sequential alignments). The algorithm was designed so as to identify the best structural alignment between protein pairs by disregarding the connectivity between secondary structure elements (SSE). One of the key feature of the algorithm is utilizing the multiple vector representation for each SSE, which enables us to correctly treat bent or twisted nature of long SSE. We compared MICAN with other 9 publicly available structure alignment programs, using both reference-dependent and reference-independent evaluation methods on a variety of benchmark test sets which include both sequential and non-sequential alignments. We show that MICAN outperforms the other existing methods for reproducing reference alignments of non-sequential test sets. Further, although MICAN does not specialize in sequential structure alignment, it showed the top level performance on the sequential test sets. We also show that MICAN program is the fastest non-sequential structure alignment program among all the programs we examined here.

**Conclusions:**

MICAN is the fastest and the most accurate program among non-sequential alignment programs we examined here. These results suggest that MICAN is a highly effective tool for automatically detecting non-trivial structural relationships of proteins, such as circular permutations and segment-swapping, many of which have been identified manually by human experts so far. The source code of MICAN is freely download-able at http://www.tbp.cse.nagoya-u.ac.jp/MICAN.

## Background

Protein structure comparison techniques are of great importance for inferring distant evolutionary relationships that cannot be suggested from sequence similarity alone [1,2], and finding recurrent structural motifs in proteins [3]. Because of its importance, many structural alignment algorithms have been developed [4-6]. Although there are a variety of structure alignment algorithm, most of them follow a simple sequential rule, that is, two proteins are aligned only in sequential order. Recently, however, a number of interesting examples that show non-sequential structure similarity have been reported, where non-sequential structural similarity is the structural similarity in which structurally equivalent regions are aligned in different order in the sequence of the compared proteins. The most populated class of non-sequential structural similarity is circular permutation [7-9]. Although not as many as that of circularly permutation, the number of examples that show non-sequential similarity beyond circular permutation proved to be also significant [10]. Further, Ilyin *et al.* found that non-sequential structural alignments between proteins are not limited to proteins of any particular fold, and they are systematic and widespread across the protein universe, suggesting generality and importance of non-sequential structural relationship [10].

Non-sequential structural similarities are intriguing in terms of evolution. Currently, some examples of protein pairs with evidence for evolutionary relationships that fold into topologically different but share the same secondary structure packing arrangement have been known. One of the most interesting examples of such protein pairs is that of KH domains of hnRNP K (PDB entry 1KHM) and ribosomal protein S3 (PDB entry 1J5E) [11]. They show statistically significant sequence similarity (38% sequence identity) implying homology. Their structures, however, are quite different in topology while converging to the same architecture. Another example is a pair of Carboxylic esterase (PDB entry 1YAS) and Hydroxynitrile lyase (PDB entry 1QLW) [10]. Both belong to the same superfamily (alpha/beta-Hydrolases superfamily) defined in SCOP database [12], suggesting that they share a common evolutionary ancestor. However, structure alignment revealed that the sequence order of the long structurally equivalent region is swapped [10]. These examples suggest that some proteins have changed their folds by segment shuffling or rearrangement events while conserving the same core packing arrangements. Further investigation of such protein pairs would give us a hint as to how proteins can change their fold in the course of evolution.

**Figure 1:**
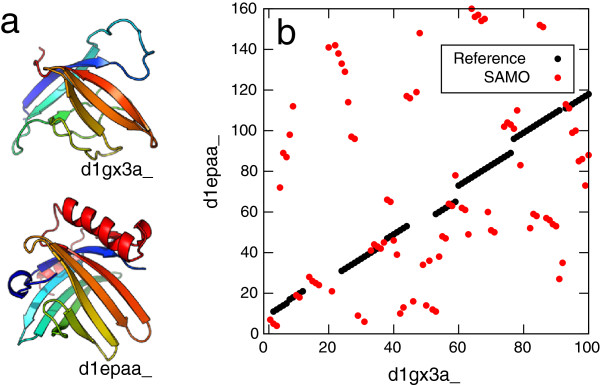
**The illustrative example of ‘over-fragmentation’ in alignment.** The comparison of a manually curated structure alignment with an automatically generated alignment by a non-sequential structure alignment program, SAMO. (**a**) The structures of arylamine N-acetyltransferase (SCOP-ID d1gx3a_, shown on upper part) and epididymal retinoic acid-binding protein (SCOP-ID d1epaa_, shown on lower part). This pair is taken from MALISAM database. (**b**) displays the alignment plot of the pair. Black points indicate the reference alignment and red indicate alignment by SAMO. The reference alignment shows RMSD of 4.4 Å over 77 residues, while the alignment by SAMO gives 3.4 Å over 91 residues.

Although some non-sequential structure alignment methods have been developed [13-22], there is still much room for improvement in non-sequential structure alignment algorithm. Indeed, we have tested all of the publicly available non-sequential structure alignment programs, and found out some problems of currently existing methods which is specific to non-sequential structure alignment. One of the problems is that some non-sequential structure alignment algorithms which utilize a single vector representation of each SSE tend to fail in aligning residues which belong to long SSEs. Such a simplified protein representation is crucial especially for non-sequential alignment, because search space for non-sequential alignment is much larger than that for sequential alignments. However, such an approach is not suitable for describing bent or twisted nature of long SSEs, which makes it difficult to correctly align long SSE regions. Also, it may be difficult to correctly align a short SSE in a long one by the single vector representation, even if the long SSE is not bent or twisted. On the other hand, another kind of the non-sequential structure alignment method which employ a C_*α*_ representation of protein structures has another kind of problem. For instance, alignments generated by SAMO [18], which tries to maximize the number of matching C_*α*_ atoms and minimize their root mean square distance, frequently show over-fragmentation; there is no long continuous alignment region, although the number of matching residue is large and RMSD of aligned residues is small. An example of such alignment is shown in Figure 1 for arylamine N-acetyltransferase and epididymal retinoic acid-binding protein. These two structures are very similar, as is suggested by the reference alignment which is taken from MALISAM database [23], a manually curated database of structurally analogous pairs. For the same protein pair, SAMO generates a totally different alignment. The reference alignment has RMSD of 4.4 Å over 77 residues, while the alignment by SAMO gives 3.4 Å over 91 residues, suggesting alignment by SAMO is better than reference just in terms of these values. However, it is questionable whether such a highly scattered and fragmented alignment is evolutionary or physically meaningful.

In this paper, we introduce a novel non-sequential structure alignment method called “MICAN” that overcomes above-mentioned problems. MICAN is designed so as to find the rotational matrix that maximize a structural similarity score of SSE region, regardless of chain connectivity. Since MICAN utilizes an SSE as a structure unit in structure comparison, it is free from over-fragmentation problem, or noisy alignments which mostly consist of relatively short segments or single residues. The search scheme of MICAN is based on the geometric hashing paradigm. It was originally developed for model-based object recognition problems in the area of computer vision, and has been widely applied to protein structure comparison studies [13,24-27]. Geometric hashing has the property that it does not use the sequential order of the points to be compared. Therefore, it is highly suitable for structure comparison in cases where the sequential order should be ignored. One of the key feature of the algorithm is utilizing the multiple vector representation for each SSE. This feature enables us to correctly treat bent or twisted long SSEs. Another feature is a building way of reference frame in the geometric hashing technique, in which one reference frame is defined per point to be compared, not defined per pair or per triplet of points. By virtue of this technique, we can reduce the number of reference frame systems to be compared to O(n), while that of the cases in which one reference frame is defined per triplet is $O(n^{3})$, where *n* is the number of points to be compared. As a result, MICAN algorithm is very fast; it is the fastest non-sequential structure alignment program among all the programs we examined here.

In order to assess the performance of the algorithm, we compare MICAN with other publicly available non-sequential alignment programs: DEDAL, SNAP, GANGSTA+, SCALI, MASS, and SAMO, as well as sequential ones: DaliLite, CE, and TM-align. We evaluate those programs using both reference-dependent and reference-independent evaluation methods on both sequential and non-sequential benchmark test sets. We show that MICAN outperforms the other existing methods for reproducing reference alignments of non-sequential test sets. Furthermore, although MICAN does not specialize in sequential structure alignment, it shows the top level performance on the sequential test sets for both reference-dependent/independent measure.

The source code of MICAN is freely download-able at http://www.tbp.cse.nagoya-u.ac.jp/MICAN.

## Results and discussion

### Evaluation

In order to assess the performance of alignment methods as objectively as possible, we use both reference-dependent and reference-independent evaluation methods.

Reference-dependent evaluation is based on pre-computed gold standard of reference alignments. Reference alignments are often carefully inspected, or manually curated to ensure good alignment quality. They are considered to be, for the most part, biologically or physically meaningful, although they may not be necessarily perfect. The reference-dependent alignment accuracy is calculated as the number of correctly aligned residue pairs in a test alignment divided by the total number of aligned residue pairs in a reference alignment. Because of its biological or physical relevance, as well as simplicity of the evaluation, reference-dependent evaluation has been widely used [28-32]. However, some aspects of the method have been criticized; it relies heavily on the correctness of the reference alignment and it is unclear how the quality of reference alignment influence the evaluation of alignment programs [6,31,33].

On the other hand, reference-independent method does not require reference alignments. It evaluates purely geometric measure, such as number of aligned residue pairs, their RMSD, number of gap, or their combinations. Thus, reference-independent method is free from problems associated with reference-dependent evaluation. Comparing these geometric measure is particularly useful for identifying characteristic feature of several alignment programs. However, these similarity measures may not necessarily reflect the biologically or physically-meaningful similarity [34]. This situation is particularly serious, especially for non-sequential structure alignment. For instance, as shown in Figure 1, an alignment by SAMO shows larger number of aligned residue pairs and lower RMSD than those of the reference alignment. However, its alignment seems to be not physically relevant because there are too many gaps and too many short segments that are likely to be spurious matches in the alignment.

As mentioned above, both reference-dependent and reference-independent method have their own advantages and weaknesses. The use of both two evaluation methods would provide more objective view on the results of the benchmark test, highlighting different aspects of the algorithm.

### Datasets

What kind of datasets should we use for the evaluation of non-sequential structural alignment programs? Obviously, many protein pairs of which the ‘correct’ alignments is known to be non-sequential are needed. We refer to such test sets as ‘non-sequential test sets’. In addition to that, ‘sequential test sets’, a collection of protein pairs of which the ‘correct’ alignment is sequential, are also needed. That is because ideally, if the correct alignment is sequential, non-sequential alignment programs should generate a sequential alignment. Sequential test sets enable us to assess such an ability of non-sequential programs. Thus, we have used both sequential and non-sequential test sets for the evaluation of different structural alignment programs.

As sequential test sets, we chose MALIDUP [35] and MALISAM [23], which contains manually curated structural alignments with non-trivial homology (MALIDUP) or structural analogy (MALISAM). We call these benchmark sets as “MALIDUP-sq” and “MALISAM-sq”, hereafter.

We must construct non-sequential test sets for evaluation of non-sequential alignment, because of the absence of gold standard of non-sequential structural alignment databases. Here, we create artificial non-sequential test sets “MALIDUP-ns” and “MALISAM-ns” based on the sequential test sets, rather than collecting naturally occurring non-sequential alignments, because the number of currently known naturally occurring protein pairs that show non-sequential structural similarity is limited. The detailed description of how to create artificial non-sequential test sets is given in the Methods section.

### Comparison to other alignment programs

We compared the alignments of MICAN with those of other structure alignment programs. They are three sequential alignment programs: DaliLite [36], TM-align [37], and CE [38], and six non-sequential alignment programs: DEDAL [33], SNAP [22], GANGSTA+ [21], MASS [13], SCALI [16], and SAMO [18]. We selected DaliLite, TM-align and CE as representative of sequential alignment programs, because they have been known to be one of the best structure alignment programs [4,6,29]. The choice of above-mentioned non-sequential programs was based on the availability of stand-alone programs. All of the programs were downloaded and executed locally on a Linux platform. If the source code of the program was available, we compiled it with GNU Compiler 4.5.5 on the computer. To our knowledge, this is currently the largest and most comprehensive comparison of non-sequential structural alignment programs.

### Benchmark test with reference-dependent evaluation

#### Results of sequential alignment test

**Figure 2:**
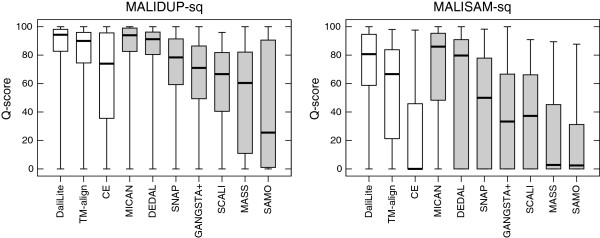
**The results of reference-dependent assessment with sequential test sets.** The box-and-whisker plot of the distribution of the Q-score obtained for three sequential (white boxes) and seven non-sequential methods (gray boxes). The left (right) plot represents the result for the test with MALIDUP-sq (MALISAM-sq) set.

**Figure 3:**
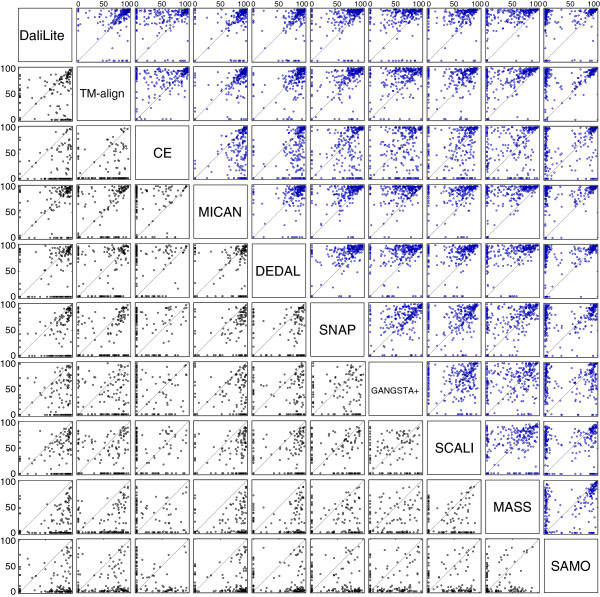
**The scatter plots of Q-score for all pairs of the compared methods for the sequential test sets.** Comparison of the Q-score for all pairs of the compared methods for the sequential test sets. Upper right diagonal shows the scatter plots of MALIDUP-sq set. Lower left shows those of MALISAM-sq set.

In order to assess alignment accuracy of several method on sequential test sets, we compared the alignments generated by the ten methods with all of the reference alignment of MALIDUP-sq and MALISAM-sq, and computed percentage of agreement, Q-score. Here, Q-score is defined as the number of correctly aligned residue pairs in a test alignment divided by the total number of aligned residue pairs in a reference alignment. The corresponding box-and-whisker plots are shown in Figure 2, and mean Q-score are listed in Table 1. We also present the scatter plots of Q-score for all pairs of the compared methods in Figure 3. For MALIDUP-sq test set, the largest mean Q-score was achieved by MICAN with 85.8% (median 94.9%) and the second largest mean was 85.3% (median 94.3%) by DaliLite. On the other hand, for MALISAM-sq test set, the best algorithm was DaliLite and the second best was MICAN. Its mean Q-score was 67.3% (median 80.7%) by DaliLite and 65.5% (median 85.9%) by MICAN. The alignment accuracy by DaliLite and MICAN are statistically indistinguishable; According to the Wilcoxon signed rank test, p-value is 0.602 and 0.937 on MALIDUP-sq and on MALISAM-sq, respectively. These two programs are the leading methods in the comparison, because the difference between these two and the others are statistically significant with *P *< 0.01. For example, the alignment accuracy by MICAN and DEDAL is statistically distinguishable; p-value is 9.7×10^−5^ and 2.7×10^−3^ on MALIDUP-sq and on MALISAM-sq, respectively. The scatter plots of Q-score shown in Figure 3 also show superiority of the two programs. From these results, it can be concluded that MICAN and DaliLite showed the best performance at least for reference-dependent evaluation on MALIDUP-sq/MALISAM-sq test sets. It should be noted that although MICAN does not specialize in sequential structure alignment, the performance of MICAN is comparable with that of DaliLite, which is known to be one of the best sequential alignment program.

**Table 1:** Average Q-scores by ten different algorithms

**Dataset**	**DaliLite**	**TM-align**	**CE**	**MICAN**	**DEDAL**	**SNAP**	**GANGSTA+**	**SCALI**	**MASS**	**SAMO**
MALIDUP-sq	85.3	81.0	62.3	**85.8**	83.4	70.8	64.4	56.2	50.4	42.8
MALISAM-sq	**67.3**	53.7	21.2	65.5	56.2	43.4	36.0	35.8	21.3	17.0
MALIDUP-ns	37.9	43.0	—	**85.6**	81.4	71.3	63.3	54.2	51.5	19.0
MALISAM-ns	21.7	22.5	—	**67.6**	56.1	43.6	29.9	37.5	20.4	8.5

#### Results of non-sequential alignment test

**Figure 4:**
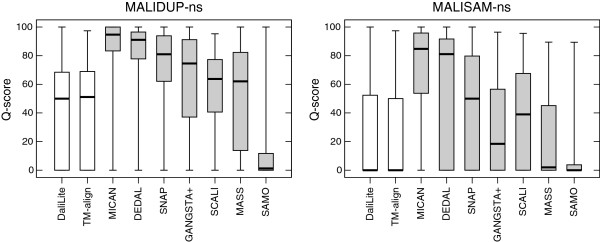
**The results of reference-dependent assessment with non-sequential test sets.** The box-and-whisker plot of the distribution of the Q-score obtained for three sequential (white boxes) and seven non-sequential methods (gray boxes). The left (right) plot represents the result of the test with MALIDUP-ns (MALISAM-ns) set.

**Figure 5:**
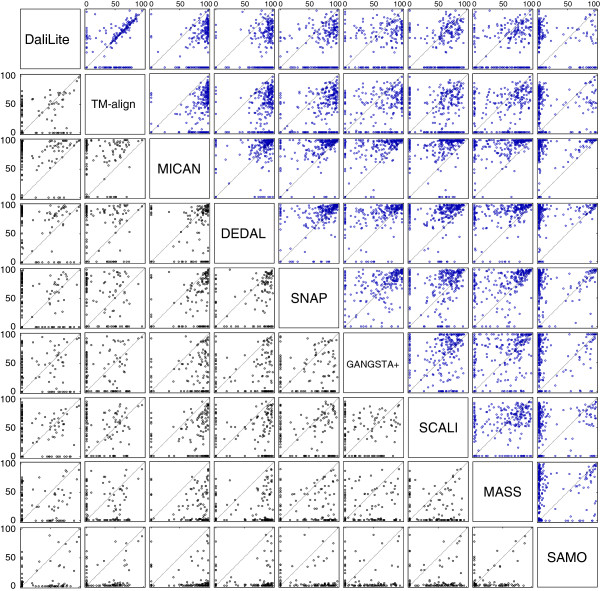
**The scatter plots of Q-score for all pairs of the compared methods for the non-sequential test set.** Comparison of the Q-score for all pairs of the compared methods for the non-sequential test sets. Upper right diagonal shows the scatter plots of MALIDUP-ns set. Lower left shows those of MALISAM-ns set.

To examine the ability of reproducing non-sequential reference alignments, we performed the benchmark test on the non-sequential test sets, MALIDUP-ns and MALISAM-ns test set. As far as we tested, CE program did not work for protein pairs in the non-sequential test sets. That is because artificially generated protein structures included in the test sets have chain break. Accordingly, data of CE algorithm is not shown for the test sets. The results of the non-sequential benchmark tests are shown in Figure 4, Figure 5, and Table 1. As we expected, the performance of sequential alignment programs, DaliLite and TM-align, were significantly decreased on non-sequential test sets, compared with their results on sequential test sets. For example, the mean Q-score obtained by DaliLite has dropped from 85.3% (MALIDUP-sq test) to 37.9% (MALIDUP-ns test). This result is a natural consequence, because these sequential alignment programs were not designed so as to align protein structures in non-sequential manner. On the other hand, the performance of non-sequential alignment programs are robust against changes of test sets from sequential to non-sequential. Among them, MICAN shows outstanding performance on the test sets. It achieves the highest median and mean Q-score on both non-sequential test sets (See Figure 4, Figure 5 and Table 1). Especially, the result on MALISAM-ns test, which is the harder one, is significant; the mean Q-score obtained by MICAN with 67.6% is as many as 11% higher than that by DEDAL, which shows the second highest mean Q-score (See Table 1). In addition, the difference between MICAN and DEDAL is statistically significant: p-value is 1.7×10^−4^ (See also Figure 4). Taken together with the results on sequential and non-sequential test set, MICAN can be considered as the outstanding program for generating alignments consistent with reference alignments regardless of whether the reference alignment is sequential or not, at least for the test sets we used here.

**Figure 6:**
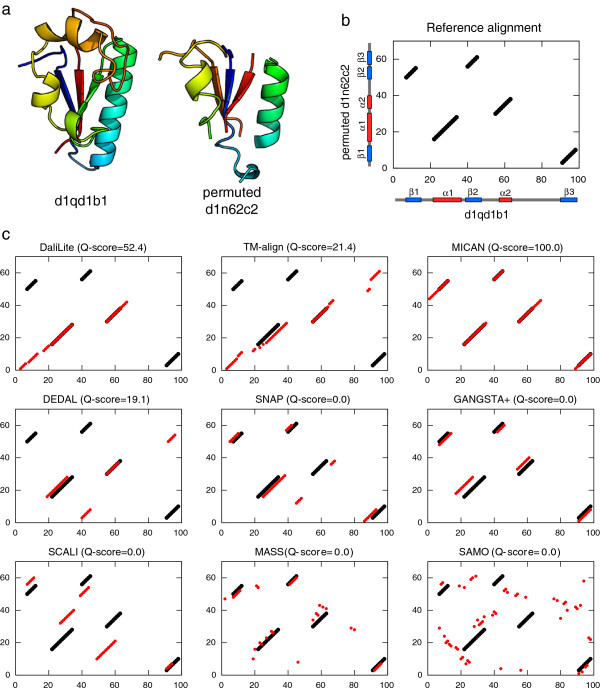
**The example of difficult non-sequential alignment taken from MALISAM-ns set.** (**a**) The structure of Formiminotransferase domain of formiminotransferase-cyclodeaminase (SCOP-ID d1qd1b1, B2083-B2180) and the permuted structure of FAD-binding domain (SCOP-ID d1n62c2, C1-C61). (**b**) The alignment plot of the reference alignment. The horizontal axis and the vertical axis correspond to residue positions of d1qd1b1 and those of permuted d1n62c2, respectively. Positions of *α* -helices and *β* -strands in the native structure are also indicated by red and blue bars. (**c**) Comparison of the agreement between the reference alignment and nine alignment methods we tested here. The name of method and its Q-score for the protein pair is shown on each the alignment plot. The reference alignment pairs are shown in black circles. Alignment pairs generated by each methods are shown in red circles.

An example of one of the most difficult alignment taken from MALISAM-ns test set is shown in Figure 6. One of the pair is the structure of Formiminotransferase domain of formiminotransferase-cyclodeaminase (shown in Figure 6a left). The other is the permuted structure of FAD-binding domain (Figure 6a right), which is generated by randomly permuting sequential order of SSEs of FAD-binding domain.

The alignment plot of the reference alignment and those of nine alignment methods are also shown in Figure 6b and c. The structure alignment of the reference consists of five fragments, and each fragment contains a single SSE and loops attached to the SSE. The orders of SSEs in the polypeptide chains are completely different, which makes alignments quite complex, as shown in Figure 6b. Five non-sequential methods (SNAP, GANGSTA+, SCALI, MASS, and SAMO) returned zero agreement with the reference alignment, suggesting difficulty of reproducing the reference alignment of this pair. DaliLite, TM-align, and DEDAL returned partial agreement with the reference. All these three method correctly aligned *α* 2 to *α* 2, but only DaliLite did *α* 1 to *α* 1. All the method except MICAN failed to correctly align any *β* strands pairs. On the other hand, only MICAN correctly align all of the secondary structure pairs, completely reproducing the reference alignment (Q-score = 100%).

**Figure 7:**
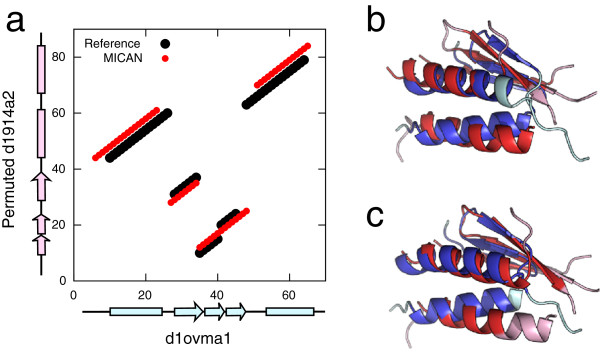
**The example of failed alignment of MICAN.** (**a**) Comparison with the reference alignment (black) and MICAN (red). This protein pair (d1ovma1 and permuted d1914a2) was taken from MALISAM-ns. The horizontal axis and the vertical axis correspond to residue positions of d1ovma1 and those of permuted d1914a2, respectively. Positions of *α* -helices and *β* -strands in the native structure are also indicated by cyan and pink bars. (**b**) The superimposition according to the reference alignment. The structure of d1ovma1 is colored in blue and cyan, and that of permuted d1914a2 is colored in red and pink. Blue and red regions indicate aligned residues, and cyan and pink represent unaligned residues. (**c**) The superimposition based on the alignment by MICAN.

Although MICAN is the best aligner in terms of reference-dependent evaluation, there are still some failures. For example, as depicted in Figure 3 and 5, MICAN generates some alignments with Q-score = 0%. Manual investigation revealed that majority of such failed alignments had a common feature; They had 1-4 residue shift of an alignment with respect to the reference alignments. Shown in Figure 7 is a typical failed example of MICAN. Although the alignment plot by MICAN is similar to that of the reference, there is no overlap with the reference alignment. TM-score of the alignment by MICAN (0.503) is slightly larger than that of the reference (0.498), indicating that optimizing TM-score does not always lead to the manual alignment. This observation suggest that one possible improvement is to develop a scoring function that is more consistent with human expert knowledge.

### Benchmark test with reference-independent evaluation

**Figure 8:**
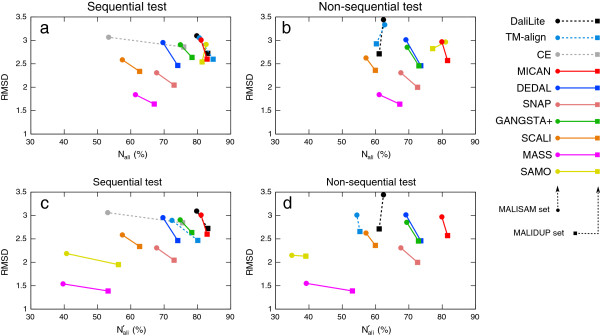
**The results of reference-independent assessment.** Results of reference-independent assessment of structural alignments are plotted on (*N*_ali_, RMSD) plane for all the tests (lines to guide the eye). X-axis and y-axis indicate the normalized number of aligned residues and its RMSD, respectively The line color represents program: black(DaliLite), cyan(TM-align), gray(CE), red(MICAN), blue(DEDAL), salmon(SNAP), green(GANGSTA+), orange(SCALI), purple(MASS), and yellow(SAMO), and the line style represents a type of the program: dotted lines represent sequential alignment programs, and normal lines non-sequential alignment programs. The benchmark sets are represented by the symbols: squares(MALIDUP-sq/ns) and circles(MALISAM-sq/ns). (**a**) and (**b**) show relationship between *N*_ali_ and RMSD for sequential and non-sequential test sets, respectively. (**c**) and (**d**) show relationship between $N_{\text{ali}}^{\prime }$ and RMSD for sequential and non-sequential test sets, respectively.

To identify characteristic feature of several alignment programs, we calculated several standard geometric measures, the number of aligned residue pairs (*N*_ali_), the corresponding root mean square deviation (RMSD) of the C_*α*_ atoms, and the number of gap opening (*N*_gap_), for all the alignments generated by all the methods, for all the pairs provided in MALIDUP-sq, MALISAM-sq, MALIDUP-ns and MALISAM-ns test sets. Here, *N*_ali_ is defined as the number of aligned residue pairs divided by the size of the smaller protein of the protein pair. Their average values for MALIDUP-sq, MALISAM-sq, MALIDUP-ns and MALISAM-ns test sets are listed in Table 2. To visualize the data, average *N*_ali_ and RMSD obtained by each method is plotted on (*N*_ali_, RMSD) plane in Figure 8. On this plane, better performance corresponds to larger values along the horizontal axis with smaller values along the vertical axis.

**Table 2:** Quality assessment by 3 reference-independent measures

**Dataset**	**Measure**	**DaliLite**	**TM-align**	**CE**	**MICAN**	**DEDAL**	**SNAP**	**GANGSTA+**	**SCALI**	**MASS**	**SAMO**
	*N*_ali_	83.5	**85.1**	76.5	82.9	74.1	73.3	78.7	63.0	67.4	81.8
MALIDUP-sq	$N_{\text{ali}}^{\prime }$	83.5	80.5	76.2	82.9	74.1	73.3	78.7	63.0	53.9	57.9
	RMSD	2.73	2.61	2.87	2.60	2.46	2.05	2.65	2.35	**1.65**	2.55
	*N*_gap_	5.5	8.8	3.9	4.1	4.0	5.0	3.2	4.2	18.6	32.5
	*N*_ali_	80.4	81.2	54.1	81.0	69.7	68.3	75.5	58.0	62.0	**83.2**
MALISAM-sq	$N_{\text{ali}}^{\prime }$	80.4	73.0	53.8	81.0	69.7	68.3	75.5	58.0	40.2	41.7
	RMSD	3.12	3.07	3.09	3.01	2.95	2.32	2.93	2.60	**1.85**	2.93
	*N*_gap_	4.8	9.5	2.3	3.6	3.8	4.6	3.4	3.5	17.4	33.4
	*N*_ali_	61.4	60.5	—	**81.6**	73.6	72.9	73.3	60.2	67.6	77.5
MALIDUP-ns	$N_{\text{ali}}^{\prime }$	61.4	55.5	—	81.6	73.6	72.9	73.2	60.2	53.8	41.2
	RMSD	2.73	2.94	—	2.57	2.46	2.01	2.47	2.37	**1.65**	2.84
	*N*_gap_	3.4	7.6	—	4.0	4.0	5.2	3.4	4.1	18.9	42.1
	*N*_ali_	63.0	63.2	—	79.9	69.1	68.2	70.0	57.6	61.7	**81.6**
MALISAM-ns	$N_{\text{ali}}^{\prime }$	63.0	54.8	—	79.9	69.1	68.2	69.9	57.6	39.7	35.4
	RMSD	3.47	3.36	—	2.97	3.01	2.32	2.87	2.64	**1.86**	2.99
	*N*_gap_	3.5	8.1	—	3.7	3.8	4.7	3.3	3.6	17.5	36.8

Shown in Figure 8a are results for MALIDUP-sq and MALISAM-sq test sets. Each method locates in various positions of the plane, which reflect the feature of objective function of each method. Among them, DaliLite, TM-align, MICAN, and SAMO show similar feature of the alignments; they are located on a similar position. These four algorithms are seated on the upper right part of the graph, suggesting that they prefer larger number of aligned residue pairs with larger RMSD (about 3 Å). Although their average RMSD values are relatively large compared with the other programs, these values can be considered as biologically relevant, because they are comparable to typical RMSDs for homologous proteins [39]. In contrast, alignments by MASS shows shorter alignment with the smallest RMSD of all the algorithm. The other programs lie between the two extreme cases.

Figure 8b shows results on MALIDUP-ns and MALISAM-ns test sets. Compared with Figure 8a, as we expected, *N*_ali_ of sequential algorithms significantly decreased. On the other hand, the positions of all of the non-sequential algorithms on the plain are not affected by the change of test sets from sequential to non-sequential test sets, compared with those of sequential algorithms. Especially, MICAN, DEDAL, SNAP and MASS are shown to be highly robust against shuffling of sequence order. Their change in the *N*_ali_ and RMSD are less than 2%.

According to Figure 8a and b, MICAN and SAMO show similar behavior; They prefer large number of aligned residue pairs with larger RMSD, regardless of whether the reference alignment is sequential or not. However, they showed totally different behavior in the reference-dependent assessment, as shown in Figure 2 and Figure 4. Where does the discrepancy come from? One possible explanation is that the large number of aligned residue pairs by SAMO is mainly attributed to contribution of short segments that are likely to be spurious matches. In fact, Table 2 shows that the number of gap opening is different between the two algorithms. The number of gap opening by SAMO is about 9 times larger than that by MICAN, suggesting that alignments by SAMO largely consist of relatively short segments or single residue pairs. As pointed out by Abyzov and Ilyin [10] and Teichert *et al.*[29], these short aligned segments are considered as ‘noise’, which may likely arise by chance, rather than ’signal’ of structural similarity, because sequence similarities based on structure alignments increase by removing short fragments from the alignments [29]. These noise could be one of the major factor of overestimation of the structural similarities [40]. Thus, in order to eliminate contribution of such noise from *N*_ali_, we calculated the number of aligned residue pairs, $N_{\text{ali}}^{\prime }$, which does not count residues pairs in fragments shorter than three consecutive residues pairs.

Average value of $N_{\text{ali}}^{\prime }$ obtained by each method for each test set are listed in Table 2. Underlined data listed in Table 2 indicate significant difference (more than 20%) between *N*_ali_ and $N_{\text{ali}}^{\prime }$. The largest change is observed for SAMO, which shows 46% decrease between *N*_ali_ and $N_{\text{ali}}^{\prime }$ on MALISAM-ns test set by omitting the short fragments, suggesting the large number of aligned residue pairs by SAMO is enhanced by gathering short (one or two residues) fragments. On the other hand, *N*_ali_ and $N_{\text{ali}}^{\prime }$ by MICAN are completely the same for all the test sets, implying that its alignments is mostly composed of relevant signals of structural similarity.

To visually understand the influence of eliminating short fragments on all the algorithms, we plotted average $N_{\text{ali}}^{\prime }$ and RMSD for each method on ($N_{\text{ali}}^{\prime }$, RMSD) plane in Figure 8c and d for sequential and non-sequential test sets, respectively. Comparing Figure 8a with c, as well as Figure 8b with d, we can see that eliminating short continuous segments bring about drastic changes for some alignment programs. As pointed out above, SAMO is the most sensitive to eliminating short fragments, followed by MASS and TM-align. The others are located at the same position on the plane, suggesting that their alignments are free from spurious matches. Among then, alignments by MICAN shows the largest number of aligned residue pairs for all the test sets, implying that the characteristic feature of MICAN is that it prefers large number of aligned residues with reasonable RMSD and that its alignment is free from spurious matches.

So far, *N*_*ali*_ and RMSD have been evaluated based on the residues located everywhere in the structure. However, as pointed out by Gueler and Knapp, comparing the number of aligned residues or RMSDs should be careful, because the residue assignment strategies can have a significant influence of the results [41]. To elucidate influence of the residue assignment strategies, we recalculated number of aligned residue pairs and corresponding RMSDs in two different strategies. The first strategy is based on the residues only within secondary structure elements(i.e. *α* -helix and *β* -strand). The second one is based on residues belonging to the same SSE type. We found that the results from both strategies were qualitatively the same as the result we have shown here; alignments by MICAN show the largest number of aligned residue pairs over all the test sets for both strategies (data now shown). This result implies the robustness of the results against the change of residue assignment strategies.

**Figure 9:**
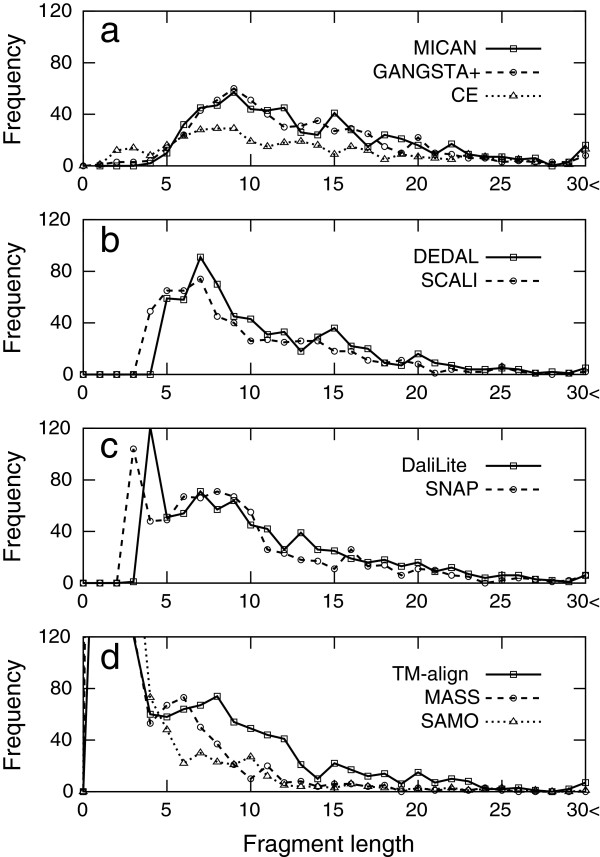
**The distributions of the length of consecutive aligned fragments for MALIDUP-sq set.** The algorithms are classified into four groups based on the location of the peak of their distribution, The first group (**a**) has a peak at 9 residue length, the second group (**b**) a peak at 7, the third group (**c**) a peak around 3 or 4, and the fourth group (**d**) a peak around 1 or 2.

We have shown that the characteristic features of alignments by MICAN are (i) large number of aligned residue pairs with reasonable RMSD and (ii) its alignment is free from one or two length short segments. For further characterization of the algorithm, we investigate what length fragments are frequently used in the alignment of MICAN, and to compare them with those of the other algorithms. Shown in Figure 9 is the distribution of lengths of continuously aligned fragments in the alignments produced by each method for MALIDUP-sq set. According to the location of the peaks of their distribution, the algorithms can be classified into four groups: The first group has a peak at 9 residue length (shown in Figure 9a), the second group at 7 (Figure 9b), the third group around 3 or 4 (Figure 9c), and the fourth group around 1 or 2 (Figure 9d). MICAN belongs to the first group; its peak location of the distribution is larger than any other groups. This result indicates that the large number of aligned residue pairs by MICAN is mostly attributed to relatively longer consecutive fragments (typically longer than 9 residue length). This is contrast to the distribution of the fourth group; The number of aligned residue pairs is enhanced by contribution of a large number of 1 or 2 length short fragments.

### Influence of multiple vector representation of protein structures on the alignment accuracy

**Figure 10:**
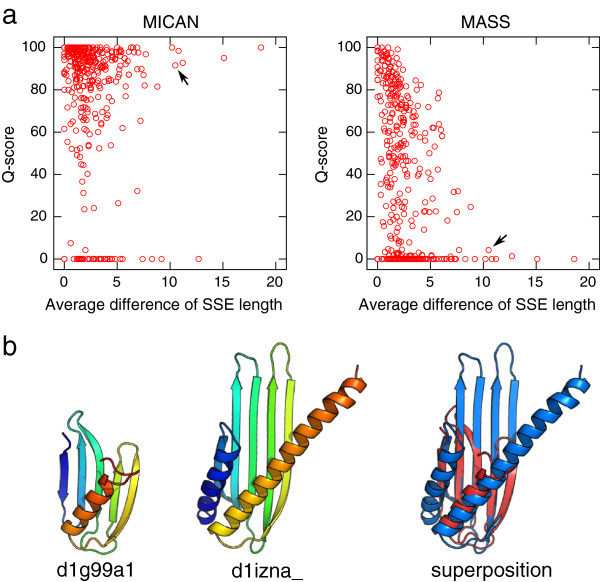
**The dependency between differences of aligned SSE length and alignment accuracy.** (**a**) Relationship between average differences of aligned SSE length and the Q-scores obtained by MICAN(left) and MASS(right). Average differences of aligned SSE length is calculated based on the reference alignment. All the targets in both MALIDUP-sq and MALISAM-sq are plotted as red circles. Black arrows indicate the protein pairs shown in (**b**). (**b**) An example of a protein pair that shows large difference of aligned SSE length. The left two cartoon structures are the target proteins, which is taken from MALISAM-sq test set. The right one is superimposition according to the reference alignment. Average difference of aligned SSE length of this pair is 11. While MASS almost failed to reproduce the reference alignment of this pair, MICAN successfully reproduced it.

As described in the Methods section, MICAN utilizes a multiple vector representation for each SSE. Here, we discuss how it works on improving performance, by comparing the performance of MICAN with that of MASS, which utilizes single vector representation of SSEs. As pointed out in the introduction, it may be difficult to correctly align a short SSE in a long one by the single vector representation. In order to assess whether the multiple vector representation improve alignment accuracy over single vector representation methods for such cases, we examined the dependence of alignment accuracy on the average difference in residue number between aligned SSEs in the reference alignment. Shown in Figure 10a is the relationship between the average difference of aligned SSE length in the reference alignment and Q-scores of MICAN and MASS alignments for all the target pairs of both MALIDUP and MALISAM-sq test set. There is statistically significant relationship between Q-score and the average difference of aligned SSE length for MASS. These two values are correlated with a correlation coefficient *r *= −0.34. The p-value associated with this correlation is extremely small (*p *= 8.0×10^−12^). This result indicates that the larger the average difference of aligned SSE length, the more MASS fail to align. On the other hand, there is no correlation between the two values of alignments by MICAN (*r *= −0.09, *p *= 0.10), implying that the performance of MICAN is robust against the difference of aligned SSE length. These results suggest that multiple vector representation of protein structures improve alignment accuracy for protein pairs that have large difference of SSE length.

### Computational costs

To assess the computation speed of the algorithms we examined here, we measured computational time per alignment. Average CPU times required to calculate one structure alignment for each test sets with the ten algorithms are shown in Table 3. The fastest algorithm of all the programs is TM-align for all the test sets. Although, compared with TM-align, MICAN is about 1.2∼2.3 times slower than TM-align, it is the fastest algorithm of all non-sequential programs. Since average CPU times required for one structure alignment by MICAN is very fast (0.097∼0.134*s*), it is suitable for large-scale database search. It should be noted that although MICAN showed the similar behavior to DaliLite in both reference-dependent/independent evaluation for sequential test sets, computational speed of MICAN is much faster (approximately 8 times faster) than that of DaliLite, which is known to be one of the best structure alignment program.

**Table 3:** Average CPU time* required to calculate one structure alignment of ten different algorithms

**Algorithm**	**MALIDUP-sq**	**MALIDUP-ns**	**MALISAM-sq**	**MALISAM-ns**
DaliLite	0.910s	1.019s	0.445s	0.539s
TM-align	**0.111s**	0.087s	**0.055s**	0.043s
CE	0.152s	—	**0.055s**	—
MICAN	**0.134s**	**0.134s**	**0.098s**	**0.097s**
DEDAL	5.734s	6.000s	0.272s	0.259s
SNAP	1.517s	1.743s	0.962s	1.128s
GANGSTA+	0.270s	0.273s	0.185s	0.190s
SCALI	8.272s	8.153s	2.723s	2.870s
MASS	0.138s	0.136s	0.105s	0.106s
SAMO	0.454s	0.455s	0.166s	0.163s

^*^CPU times are measured on a PC with Intel Core-i7 2.93 GHz processor.

### Homologous protein pairs that have non-sequential structural similarity

Recently, some homologous protein pairs that show non-sequential structure similarity have been reported [10,11]. However, it is unclear how abundant such pairs are. In order to address the issue, we compared homologous protein structures by MICAN. We considered all the pairs of SCOP family representatives within the same superfamily (12,603 pairs). For these 12,603 pairs, pairwise structural alignments were generated using MICAN. Here, only the alignments with significantly high structural similarity, TM-score ≥ 0.5 [42], were collected, resulting in total of 8,335 structurally similar protein pairs. Among them, the overall proportion of protein pairs that show non-sequential alignments was 39% (3,284 pairs). This result suggests that a large number of proteins have changed their folds by some complex events while conserving the same core packing arrangements.

**Figure 11:**
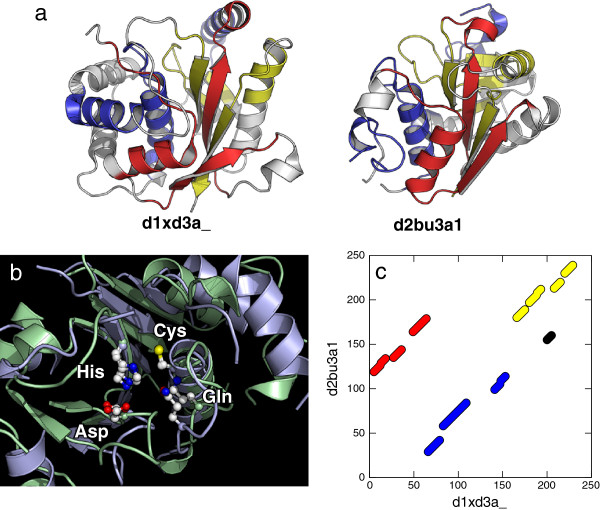
**Comparison of UCH-L3 and Phytochelatin synthase.** (**a**) The cartoon models of UCH-L3 (d1xd3a_) and Phytochelatin synthase (d2bu3a1). The aligned regions are shown in three colors red, blue and yellow corresponding to the colors used in (**c**). (**b**) A close view of the catalytic-site of the superimposed structure by MICAN. The aligned regions of UCH-L3 and Phytochelatin are shown as cartoon models in light blue and light green, respectively. The ball-and-stick representation indicate sidechain atoms of catalytic-site residues His, Asp, Cys and Gln. White, blue, red and yellow balls correspond to carbon, nitrogen, oxygen and sulfur atoms. (**c**) The structural alignment of the two proteins generated by MICAN.

One of the interesting example of such a homologous protein pair is shown in Figure 11 for Ubiquitin C-terminal hydrolase (SCOP ID d1xd3a_) and Phytochelatin synthase (SCOP ID d2bu3a1). These two proteins belong to the same superfamily (Cysteine Proteinase superfamily) in the SCOP database [12], suggesting that they share a common evolutionary ancestor. Although their ligands are different, the molecular functions are suggested to be similar; both of them have hydrolase enzymatic activity and contain the same catalytic residues of cysteine, histidine, aspartate and glutamine [43,44]. TM-score of the alignment by MICAN is 0.517 (RMSD of 3.8 Å over 70% residues), suggesting significant structural similarity. Figure 11b shows superimposed structures of aligned region around the catalytic-site. All the side-chain atoms of the catalytic residues are well superimposed, suggesting the correctness of the alignment. The corresponding alignment plot (Figure 11c) shows a non-sequential alignment; the large portion of structurally equivalent parts are not in the same order in protein sequences. This permutation can not be explained by well-established mechanism of protein evolution such as “Duplication and Fusion”. It is interesting to investigate how such a fold change occurred, but left for future studies.

## Conclusions

We have developed a new algorithm MICAN to compare protein structures regardless of the topology. One of the key of the algorithm is a novel representation of SSEs with multiple comparing elements of short segments. It enables us to properly describe bent or twisted nature of long secondary structure element. In addition, this representation method also helps speeding up a geometric hashing procedure. As a results, MICAN is the fastest program among non-sequential alignment programs we examined here.

We have presented a comparative analysis of structural alignments using both reference-dependent and reference-independent evaluation methods on both sequential and non-sequential test sets. Reference-dependent evaluation shows MICAN outperforms the other existing methods for reproducing reference alignments of non-sequential test sets. Further, although MICAN does not specialize in sequential structure alignment, its performance on sequential test sets is comparable to DaliLite, which is known to be one of the most accurate sequential alignment program. Reference-independent evaluation demonstrates that the features of alignment by MICAN are large number of aligned residue pairs with reasonable RMSD and that its alignment is free from short segments that are likely to be spurious matches. These results suggest that MICAN is highly effective tool for automatically detecting homologous protein structures bearing topological irregularities, such as circular permutations and segment-swapping, many of which have been identified manually by human experts so far.

## Methods

### The general strategy of the algorithm

Assume we have two protein structures, a query and a model, composed of *N* and *M* residues, respectively. The goal is to find the rotational matrix that maximizes structural equivalences between the two structures, regardless of chain connectivity. The search scheme of MICAN employs a hierarchical alignment algorithm, in which the SSEs are first aligned, and then a residue level alignment is iteratively performed.

The SSE level alignment is further divided into four steps: In the 1st step, secondary structure types are assigned for each residue of the two structures. In the 2nd part, we define “the Comparing Elements of the Short Segment (CESS)”, by which we describe the structures to be compared in the SSE level alignment. The CESS is a point which is defined per consecutive 3 residues for strands, and 6 for helices. Accordingly, the number of CESSs included in each SSE is *n *− 2 for strands, *n *− 5 for helices, where *n* is the length of the SSE. Each CESS has the information of its SSE type, the representative location of the SSE segment, the direction of the short segment of SSE from the N-terminus to the C-terminus, and the perpendicular vector to the SSE direction. Thus, each CESS has two vectors, which enables us to define a reference frame on each CESS. In the 3rd step, one reference frame is defined per CESS by exploiting the two vector each CESS possesses. In the 4th step, we use geometric hashing technique to pick up candidates of best rotational matrix that maximize non-sequential structural similarity by searching for the best matches of CESSs. Some candidates of best rotational matrix are listed and stored for the next stage.

The residue level alignment is divided into two steps: In the 1st step, we generate a residue level alignment based on the superimposition obtained by SSE level alignment. Starting from the initial superimposition, we generate one-to-one mapping of C_*α*_ atoms in step wise manner, in which we align residue pairs from pairs superimposed with smaller RMSD to larger. In the 2nd step, the refinement of alignment is performed iteratively until the similarity score converges. Finally, the best scoring alignment is selected based on TM-score like function. The details of each protocol is described in the following subsections.

### The SSE level alignment

#### The 1st step: SSE assignment

The starting points of MICAN algorithm is secondary structure assignment for all the residues of both the query and the model. For a given residue, a secondary structure type (*α* -helix, *β* -strand, or coil) is assigned by the method of Zhang and Skolnick [37], instead of DSSP [45] or STRIDE [46], because of its speed and applicability to C_*α*_ only models. It was reported that *Q*_3_ accuracy of the method was 85% with respect to the DSSP assignment. In the SSE level alignment, we use only residues that are assigned as *α* -helix or *β* -strand, and ignore the rest of the residues. We also ignore helices shorter than 6 residues and strands shorter than 3.

#### The 2nd step: defining the comparing elements of the short segment

**Figure 12:**
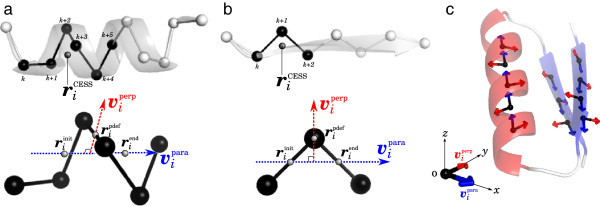
**The definition of the coordinate of CESS.** The definition of coordinate of CESS ***v***^para^, and ***v***^perp^ for (**a**) an *α* helix and (**b**) a *β* strand are depicted. In the upper parts, an *α* helix and a *β* strand are depicted by a ribbon diagram, as well as by a ball-and-stick C_*α*_ model. Black balls represents C_*α*_ atoms of *i*-th SSSSE that starts from *k*-th residue. Gray balls represents $r_{i}^{\text{CESS}}$. In the lower parts, $v_{i}^{\text{para}}$ and $v_{i}^{\text{perp}}$ of the CESS are indicated by dashed arrows colored in blue and red, respectively. (**c**) An example of schematic representation of a *β* - *α* - *β* motif by a set of CESSs.

In the second step, for both the query and the model, we define the Comparing Elements of the Short Segment (CESS), by which we describe the structures to be compared in the SSE level alignment. A CESS is defined per a Short Segment of SSE (SSSSE), where, SSSSE is defined as a segments of consecutive 3 residues for strands, and 6 for helices. Accordingly, the number of CESSs, as well as SSSSEs, included in each SSE is *n *− 2 for strands, *n *− 5 for helices, where *n* is the length of the SSE (see Figure 12a and b). Each CESS has following four kinds of information on the SSSSE. The first one is secondary structure type (i.e. *α* or *β*) which was assigned in the first step. We denote secondary structure type of *i*-th CESS as *s*_*i*_. The second one is the coordinate of the representative point of the SSSSE, which we call “the coordinate of the CESS”. We denote the coordinate of the *i*-th CESS as $r_{i}^{\text{CESS}}$. When a CESS represents the *i*-th SSSSE which starts from the *k*-th residue (see Figure 12a and b), $r_{i}^{\text{CESS}}$ is defined as the middle point of both ends of the SSSSE, i.e.

(1) $$r_{i}^{\text{CESS}}=(r_{i}^{\text{init}}+r_{i}^{\text{end}})/2,$$

where

(2) $$\begin{array}{l} r_{i}^{\text{init}}=\left\{\begin{array}{l} (0.74r_{k}+r_{k+1}+r_{k+2}\\ \;\;+0.74r_{k+3})/3.48\;\text{(for helices)}\\ (r_{k}+r_{k+1})/2\;\;\;\text{(for strands)} \end{array}\right) \end{array}$$

(3) $$\begin{array}{l} r_{i}^{\text{end}}=\left\{\begin{array}{l} (0.74r_{k+2}+r_{k+3}+r_{k+4}\\ \;\;\;+0.74r_{k+5})/3.48\;\text{(for helices)}\\ (r_{k+1}+r_{k+2})/2\;\;\;\;\text{(for strands).} \end{array}\right) \end{array}$$

Here, ***r***_*k*_ indicates coordinates of C_*α*_ atom of *k*-th residue. The expressions of $r_{i}^{\text{init}}$ and $r_{i}^{\text{end}}$ are taken from Ref. [14]. The third kind of information is the direction of the SSSSE from N- to the C-terminus. The directional vector of *i*-th SSSSE ($v_{i}^{\text{para}}$) is defined as

(4) $$v_{i}^{\text{para}}=(r_{i}^{\text{end}}-r_{i}^{\text{init}})/\left|r_{i}^{\text{end}}-r_{i}^{\text{init}}\right|,$$

which was also used in Ref. [14]. The fourth information is the perpendicular vector to $v_{i}^{\text{para}}$. The reason why we introduce this vector is that by introducing the vector we can define the reference frame per CESS. This technique enables us to reduce complexity of Geometric Hashing algorithm to $O(n^{2})$, while that of the naive geometric hashing, in which reference frame is defined per triplet of points, is $O(n^{4})$. The perpendicular vector of *i*-th SSSSE, $v_{i}^{\text{perp}}$, is defined as a normal vector of $v_{i}^{\text{para}}$ that runs through a point $r_{i}^{\text{pdef}}$, where $r_{i}^{\text{pdef}}$ is, roughly speaking, the location of the SSSSE center, and defined as

(5) $$r_{i}^{\text{pdef}}=\left\{\begin{array}{ll} (r_{k+2}+r_{k+3})/2 & \text{(for helices)}\\ r_{k+1} & \text{(for strands).} \end{array}\right)$$

In the same way, we calculate above mentioned quantities for every SSSSE. As a result, whole structures of both the query and the model are described by a set of CESSs. An example of schematic representation of a protein structure by a set of CESSs is shown in Figure 12c.

#### The 3rd step: defining the reference frame

In this step, we define reference frames for each CESS of both the query and the model. Reference frames are coordinate systems, which are used in geometric hashing technique. As described in the 2nd step, each CESS has two vectors, which are orthogonal to each other. By the use of two vectors, a coordinate reference frame for *i*-th CESS is defined as follows. The origin of the reference frame is placed on the coordinate of *i*-th CESS. The *x*-axis is defined by the vector $v_{i}^{\text{para}}$ and *y*-axis is $v_{i}^{\text{perp}}$. The *z*-axis is defined to be perpendicular to the *x*-*y* plane and its direction is determined by the right-handed rule.

#### The 4th step: identifying candidates of best rotational matrix by geometric hashing

In order to find candidates of best rotational matrix, which maximizes coincidences of CESSs of the two structures, we use geometric hashing technique. Geometric hashing algorithm is composed of two interrelated phases: preprocessing and recognition.

In the preprocessing phase, we set up hashing table for the model structure. The preprocessing procedure we use here is essentially the same as the standard one, except for a set of descriptions stored in the hash table. For each reference frame, we define 3D grid. A resolution of the grid is set to 3.2 Å in the algorithm. The cells of the grid make up a hash table, which we denote as *H*(*a*,*b*,*c*), where (*a*,*b*,*c*) is an index of 3D cube. First, *i*-th CESS in the model is chosen as a basis of the reference frame. Second, the position of *j*-th CESS in the reference frame *i*, which we call $r_{j}^{\text{CESS}}(i)$, is calculated according to the formula, $r_{j}^{\text{CESS}}(i)\equiv [(r_{j}^{\text{CESS}}-r_{i}^{\text{CESS}})\cdot {\hat{x}}_{i},(r_{j}^{\text{CESS}}-r_{i}^{\text{CESS}})\cdot {\hat{y}}_{i},(r_{j}^{\text{CESS}}-r_{i}^{\text{CESS}})\cdot {\hat{z}}_{i}]$, where ${\hat{x}}_{i}$, ${\hat{y}}_{i}$, and ${\hat{z}}_{i}$ are base vectors for *x*, *y*, and *z* axis of the reference frame *i*, and $r_{i}^{\text{CESS}}$ and $r_{j}^{\text{CESS}}$ are the coordinates of *i* and *j*-th CESS in the original coordinate system. Third, if $r_{j}^{\text{CESS}}(i)$ is found in the cube (*a*,*b*,*c*), following four descriptions of *j*-th SSSSE are stored in the hash table *H*(*a*,*b*,*c*) : these are (i) an identification of its SSE type (*s*_*j*_), (ii) an identification of the basis (in this case, the CESS that defines the reference frame *i*), (iii) the transformed vector ***v***_*j*_^para^(*i*), which is the vector ***v***_*j*_^para^ calculated in the reference frame *i*, and (iv) the transformed vector ***v***_*j*_^perp^(*i*). This procedure is repeated for all the CESS of all reference frames.

In the recognition phase, we compare the model and the query structure. The goal of this step is to find candidates of the rotational matrix that maximizes coincidences of CESSs of the two structures. The recognition phase of MICAN is also essentially the same as standard geometric hashing algorithm, except for a voting score. Let *k*-th CESS of the query structure be the selected point which defines the reference frame. For each of the other CESS of the query, the coordinates in the frame system defined by the *k*-th CESS are computed, as for the model. These coordinates are used as indexes of 3D cube. If $r_{l}^{\text{CESS}}(k)$ of the query is found in the cube (*a*,*b*,*c*), and information of *j*-th CESS of the frame system *i* of the model is stored in the hash table *H*(*a*,*b*,*c*), voting score *S*(*j*(*i*),*l*(*k*)) is added for the pair of reference frames *i* and *k*. The function of the voting score *S*(*j*(*i*),*l*(*k*)) is defined as

(6) $$S(j(i),l(k))=\Theta_{j(i),l(k)}+\Phi_{j(i),l(k)},$$

where

(7) $$\begin{array}{l} \Theta_{j(i),l(k)}=\left\{\begin{array}{ll} 0 & \left(|\theta |>\Pi /3\right)\\ \frac{\cos(\theta_{j(i),l(k)})-\cos(\Pi /3)}{1-\cos(\Pi /3)} & (|\theta |\leq \Pi /3), \end{array}\right) \end{array}$$

(8) $$\begin{array}{l} \Phi_{j(i),l(k)}=\left\{\begin{array}{ll} 0 & \left(|\phi |>\Pi /3\right)\\ \frac{\cos(\phi_{j(i),l(k)})-\cos(\Pi /3)}{1-\cos(\Pi /3)} & (|\phi |\leq \Pi /3). \end{array}\right) \end{array}$$

Here, *θ*_*j*(*i*),*l*(*k*)_ and *ϕ*_*j*(*i*),*l*(*k*)_ are the angle between $v_{j}^{\text{para}}(i)$ and $v_{l}^{\text{para}}(k)$, and that between $v_{j}^{\text{perp}}(i)$ and $v_{l}^{\text{perp}}(k)$, respectively. This procedure is repeated for all the other CESS of all the reference frames of the query structure. As a result, all the reference frame pairs between the model and the query are ranked according to their summation of the voting score. Top scoring 50 reference frame pairs are stored for the next step. For each such pairs, we calculate a rotational matrix by superimposing one reference frame onto the other. These rotational matrices are used to set up an initial superimposition of the two structures in the residue level alignment.

### The residue level alignment

#### The 1st step: one-to-one assignment of C_*α*_ atoms

In this step, starting from the initial superimposition obtained by SSE level alignment, we generate one-to-one mapping of C_*α*_ atoms in a step wise manner, in which we align residue pairs from pairs superimposed with smaller RMSD to larger. In order to identify which residue pairs are superimposed with given cut off distance *d*_*R*_, we construct an *N *×* M* similarity matrix *M*_*i*,*j*_(*d*_*R*_) based on a given superimposition of the query and the model structure, where *N* and *M* denote the number of residues in the query and the model. The matrix *M*_*ij*_(*d*_*R*_) is defined as

(9) $$M_{i,j}(d_{R})=\left\{\begin{array}{ll} \frac{1}{1+d_{\text{ij}}^{2}/d_{0}^{2}}\times \frac{\delta_{\sigma_{i}\sigma_{j}}+w}{1+w} & \left(d_{\text{ij}}\leq d_{R}\right)\\ 0 & \text{(otherwise),} \end{array}\right)$$

where *d*_*ij*_ is the distance between the C_*α*_ atom of the *i*-th residue of the query and that of the *j*-th residue of the model, *d*_0_ is a scale to normalize the match difference, *d*_*R*_ is cut off distance of *R*-th step, *σ*_*i*_ represent the three-states of secondary structure (helix, strand or coil) of residue *i*, and $\delta_{\sigma_{i}\sigma_{j}}$ is Kronecker’s delta. The scale *d*_0_ is defined as $d_{0}=1.24\sqrt[{3}]{L-15}-1.8$, where *L* is the length of the query structure. This formula was originally introduced by Zhang & Skolnick, to eliminate the inherent protein size dependence of the score function [47]. The factor $\left(\delta_{s_{i}s_{j}}+w)/(1+w\right)$ was introduced so that aligned residues should belong to the same SSE type. Here, *w* is a weight factor and is set to 1.0 in this paper. The cut off values *d*_*R*_ in each step *R* are set as follows: *d*_1 _= 3.2 Å, *d*_2 _= 1.5×3.2 Å, and *d*_3 _= 2.5×3.2 Å.

We consider alignments composed only of continuous segments with certain length, in order to eliminate short segments that are likely to be spurious matches. Generally, a continuous segment of length *l* is described as a set of residue pairs; {(*α*,*β*),(*α*+1,*β*+1),⋯,(*α*+*l*−1,*β*+*l*−1)}, where (*α*,*β*) indicates that residue *α* of the query is paired with the residue *β* of the model. Here, given the cut off value *d*_*R*_, we consider only continuous segments that fulfill the condition;

(10) $$M_{\alpha +m,\beta +m}(d_{R})>0\;\text{for}\;\forall m\in (0,1,\cdots \;,l-1).$$

We refer to such a segment as *A*_*ζ*_(*d*_*R*_), where *ζ* is the index of the segment. We introduce the similarity score *S*[*A*_*ζ*_(*d*_*R*_)] of the segment *A*_*ζ*_(*d*_*R*_), which is defined as

(11) $$S[A_{\zeta }(d_{R})]=\overset{l-1}{\underset{m=0}{\sum }}M_{\alpha +m,\beta +m}(d_{R}).$$

The similarity score is used to rank the segments, as well as to exclude short segments.

First, we align residue pairs that are superimposable with the smallest cut off value, *d*_1_. We calculate *M*_*i*,*j*_(*d*_1_) based on the initial superimposition obtained by SSE level alignment, and generate alignments based on the matrix. The goal of this step is to find a set of non-overlapping continuous segments {*A*_1_(*d*_1_),*A*_2_(*d*_1_),⋯,*A*_*n*_(*d*_1_)} that maximizes

(12) $$S_{\text{tot}}(d_{1})=\overset{n}{\underset{k=1}{\sum }}S[A_{k}(d_{1})],$$

where *n* is the number of segments in the alignment. Since the exact solution of this problem is hard to obtain, our approach is to use greedy-like algorithms to find an approximate solution. First, the algorithm chooses the continuous segment that has the highest score of all the continuous segments on the matrix. We refer to such a segment as *A*_1_(*d*_1_). If *S*[*A*_1_(*d*_1_)] satisfies the condition, *S*[*A*_1_(*d*_1_)] ≥* S*_*min*_, *A*_1_(*d*_1_) is recorded as the first member of the set of best segments, where the cutoff parameter *S*_*min*_ is set to 2.2 in this paper. Then, in order to choose the next segment, we modify the matrix *M*_*i*,*j*_(*d*_1_) such that matrix elements interfering with *A*_1_(*d*_1_) are set to zero. In other words, if the selected segments *A*_1_(*d*_1_) is described as *A*_1_(*d*_1_) = {(*α*,*β*),(*α*+1,*β*+1),⋯,(*α*+*l*−1,*β*+*l*−1)}, matrix elements whose row number is *i* (*α *≤* i *≤* α *+* l *− 1) or whose column number is *j* (*β *≤* j *≤* β *+* l *− 1) are set to 0, except for *A*_1_(*d*_1_) itself. We also set matrix elements of the segment *A*_1_(*d*_1_) to zero so that we won’t select *A*_1_(*d*_1_) as a highest score segment in the following steps. Next, we choose the continuous segment that has the highest score of all the continuous segments on the modified matrix. We refer to such a segment as *A*_2_(*d*_1_). If *S*[*A*_2_(*d*_1_)] ≥* S*_*min*_, *A*_2_(*d*_1_) is recorded as the second member of the set of best segments. Further, we modify the matrix such that matrix elements interfering with *A*_2_(*d*_1_), as well as those of *A*_2_(*d*_1_), are set to zero. We repeat this procedure until the highest score of all remaining segments on the modified matrix is smaller than *S*_*min*_. As a result, we obtain a set of non-overlapping segments that approximately maximize the total score *S*_*tot*_(*d*_1_) for given cut off distance *d*_1_. We describe such a set as $\left\{A_{1}(d_{1}),A_{2}(d_{1}),\cdots \;,A_{n(d_{1})}(d_{1})\right\}$, where *n*(*d*_1_) is the number of the best segments for the given cut off value *d*_1_.

Second, we extend the alignment with increasing the cut off value *d*_*R*_ from *d*_2_ to *d*_3_ in a step wise manner. For each *R*-th step, as in the case of *d*_1_, we calculate the similarity matrix *M*_*i*,*j*_(*d*_*R*_) according to the equation (9). We modify the matrix *M*_*i*,*j*_(*d*_*R*_) such that matrix elements interfering with any of the *A*_*i*_(*d*_*R*−1_) are set to zero, where *A*_*i*_(*d*_*R*−1_) is a segment involved in the best alignment obtained in (*R*−1) -th step. This operation assures that the matrix elements of the best segments identified in (*R*−1) -th step are included in the best segments of *R*-th step. Then, the algorithm chooses the segment that has the highest score of all the segments on the modified matrix. It is recorded as the first member of the best segments in *R*-th step. We denote such a segment as *A*_1_(*d*_*R*_). Next, we further modify the matrix such that matrix elements interfering with *A*_1_(*d*_*R*_), as well as those of *A*_1_(*d*_*R*_), are set to zero. We repeat these procedures until the highest score of all remaining segments on the modified matrix is smaller than *S*_*min*_, for given cut off *d*_*R*_. The resultant best set of non-overlapping segments for *d*_*R*_ is described as $\left\{A_{1}(d_{R}),A_{2}(d_{R}),\cdots \;,A_{n(d_{R})}(d_{R})\right\}$, where *n*(*d*_*R*_) is the number of the best segments for the given cut off value *d*_*R*_.

By repeating the same procedure from *d*_2_ to *d*_3_, we finally obtain the set of non-overlapping segments for *d*_3_, {*A*_1_(*d*_3_),*A*_2_(*d*_3_),⋯,*A*_*n*_(*d*_3_)}, which is used as the initial alignment in the iterative refinement step.

#### The 2nd step: iterative refinement

The final step of the algorithm is refinement of the alignment. It is performed iteratively as follows. First, we superimpose the structures by minimizing the RMSD of C_*α*_ atoms based on the aligned residues identified in the previous step. Second, based on the superposition, we obtain a new alignment by the method as was described in the first step of the residue level alignment. These procedures are repeated until the score no longer improves. The score we use here is modified TM-score, which is defined as

(13) $$\text{mTM-score}=\frac{1}{N}\underset{\langle i,j\rangle }{\sum }M_{\text{ij}}(d_{3}).$$

Here, *N* is the length of the query, and $\underset{\langle i,j\rangle }{\sum }$ indicates that summation is taken over all of the aligned residue pairs. Finally, the alignment with the highest mTM-score is returned. The alignments usually converge in moderate time, although there is a discrepancy in the algorithm; In superimposing structures, we minimize RMSD rather than mTM-score, which is the objective function in the algorithm. As far as we have tested, the score typically converges by 5-6 iterations, suggesting that the discrepancy is not critical.

### Selecting the best alignment

Since the score function used in the SSE level alignment is different from that in the residue level, the top scoring superposition obtained in the SSE level alignment may not always lead to the best scoring alignment in the residue level. We explored the relationship between the two scoring function and found that although they did not have perfect linear relationship, there was a strong correlation between the two. Further, we confirmed that if we started from each of the top scoring 50 superimposition of the SSE level alignment, we reached the highest mTM-score in the residue level alignment in most cases. Thus, in order to obtain as high scoring an alignment of the residue level as possible, MICAN performs the residue level alignment starting from each of the top scoring 50 superposition of the SSE level alignment. An alignment with the highest mTM-score among those 50 alignments is returned as the best alignment. MICAN program can output sub-optimal alignments, if the user specifies the option of output of alternative alignments.

### Handling reverse alignments

It has been reported that there are many interesting examples of non-sequential structure alignments that involve reverse alignments, in which SSEs structurally match each other but the polypeptide chains go in opposite directions [10,21]. MICAN can deal with forward and reverse alignments simultaneously, if needed. In order to allow reverse alignments, the algorithm should be changed as follows: 

• The function Θ in equation (7) is changed as

$$\begin{array}{l} \Theta_{j(i),l(k)}=\left\{\begin{array}{ll} 0 & \left(|\theta |>\Pi /3\right)\\ \frac{\left|\cos(\theta_{j(i),l(k)})|-\cos(\Pi /3\right)}{1-\cos(\Pi /3)} & (|\theta |\leq \Pi /3). \end{array}\right) \end{array}$$

• In the residue level alignment, we consider not only forward segments but also reverse ones, which is described as {(*α*,*β*),(*α*+1,*β*−1),⋯,(*α*+*l*−1,*β*−*l*−1)}.

This algorithm is implemented in MICAN program. Users can select either the forward only or forward/reverse mixed mode. All the data shown in this paper were obtained in forward only mode.

### Parameter tuning

There are a number of parameters that need to be optimized in MICAN algorithm, such as *w* and *d*_*R*_ in equation (9). We determined these parameters by maximizing agreement with the reference alignments of SISY-pairwise dataset [30] through try-and-error procedure in a non-systematic way. The SISY-pairwise was derived from SISYPHUS database [48], a manually curated multiple structure alignment database which contains structural alignments with nontrivial relationships including circular permutation or segment swap, thus the dataset is suitable for training of non-sequential alignment. Through training, we found that some parameters have strong influence on the performance. One of such parameters is a distance cutoff value *d*_*R*_ used in the residue level alignment.

**Figure 13:**
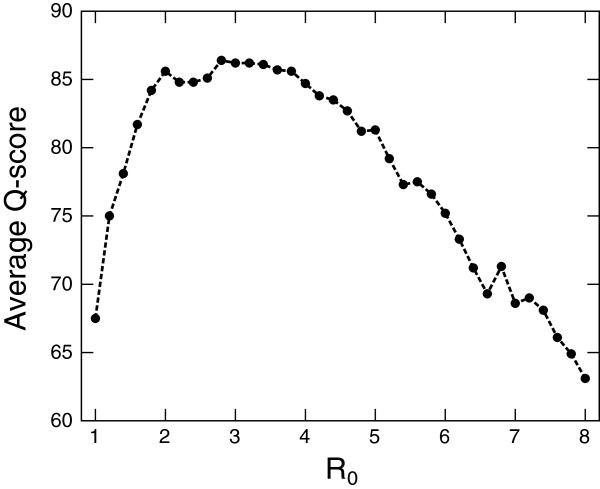
**The cutoff distance dependence of the mean Q-score for the SISY set.** The mean Q-score is calculated for several *R*_0_ values on the SISY set. All the other parameters are set to the same values as those we used in this paper.

In this work, we set *d*_*R*_ in each step *R* as *d*_1 _=* R*_0_, *d*_2 _= 1.5 ×* R*_0_ and *d*_3 _= 2.5 ×* R*_0_, where *R*_0_ is the cutoff parameter. Thus, the single parameter *R*_0_ determines the behavior of *d*_*R*_. Figure 13 shows the relationship between the mean Q-score and *R*_0_ for the SISY test set. We clearly see that the optimal value is located near *R*_0 _= 3 Å, and that the mean Q-score sharply drops as *R*_0_ value changes from the optimal point. Another is the voxel size *h* used in geometric hashing algorithm. We observed that the voxel size dependence of the mean Q-score is qualitatively similar to the case of *R*_0_. Preliminary numerical tests on a variety of *d*_*R*_ and *h* values led to the choice of *R*_0 _= 3.2 Å and *h *= 3.2 Å. The other parameters seems to be not critical for the performance. After the training, MICAN finally achieved high agreement (86.2% on average) with the reference alignments. This agreement is better than that of DaliLite (77.4% on average), which is known to be one of the best structure alignment program. We believe that the parameter set we determined is nearly optimal, although it has not been optimized systematically.

### Datasets

#### Sequential datasets

As sequential test sets, we chose MALIDUP[35] and MALISAM [23], which contains manually curated structural alignments with non-trivial homology (MALIDUP) or structural analogy (MALISAM). MALIDUP consists of 241 protein pairs and MALISAM 130 pairs. There is no overlap between the MALISAM test set and the SISY training set. On the other hand, the MALIDUP test set has the same three protein pairs used in the training set. However, these three pairs constitute only 1.2% of the MALIDUP test set. Thus, the test set can be considered as almost independent from the training set.

These two test sets are one of the most challenging benchmark sets, because they contain more difficult targets than other databases such as HOMSTRAD [49]; We performed structural alignment benchmark tests using DaliLite [36], and found that average values of fraction of correctly aligned residue pairs are 90.1%, 85.3% and 67.3% for HOMSTRAD, MALIDUP and MALISAM, respectively.

#### Non-sequential datasets

**Figure 14:**
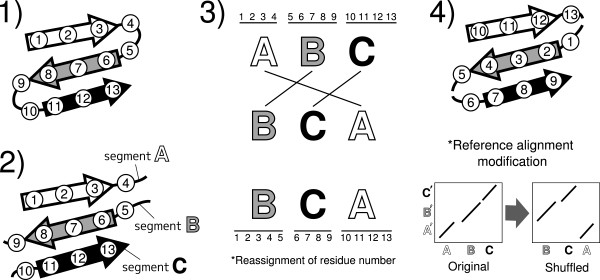
**The schematic representation of how to create a non-sequential test sets.** (**1**) One protein structure to be permuted is randomly chosen from each target pair. Original residue numbers are indicated inside circles. The three SSEs (strands) are represented by arrows. (**2**) The original chain is splitted into several segments at loop positions. In this example, the chain has been divided into three segments A, B and C. (**3**) The segment order is randomly shuffled (e.g. the order A-B-C was shuffled into different order B-C-A). Residue numbers are reassigned according to the shuffled segment order. (**4**) The generated structure by the permutation technique. Its residue numbers are also modified according to the segment order. The reference alignment are modified according to the reassigned residue numbers of the permuted protein. A’, B’ and C’ represent segments of the other protein structure which has not been chosen for sequence shuffling. Segments A’, B’ and C’ correspond structurally to segments A, B and C respectively.

We created two artificial non-sequential test sets “MALIDUP-ns” and “MALISAM-ns” based on the sequential sets, MALIDUP-sq and MALISAM-sq, by multiple segment permutations technique. This technique is also used for production of reasonable decoy sets of protein structures, conserving natural spacial arrangement of atoms [50,51]. The creation scheme is described as follows (See also Figure 14). 

(1) We chose one protein structure randomly from each target pair, and identified loop regions by DSSP program [45].

(2) The chain was splitted into several segments at all the loop regions.

(3) The linear order of these segments was randomly shuffled, and the residue numbers were reassigned accordingly. We did not perform loop modeling for those permuted structures.

(4) Reference alignment of the each target pair was modified according to the reassigned residue numbers.

In this way, we generated one non-sequential reference alignment from each sequential reference alignment. Performing the same procedure for all the sequential test sets yields 241 and 130 non-sequential alignments from MALIDUP-sq and MALISAM-sq. The sequential/non-sequential test sets we used here are available online at http://www.tbp.cse.nagoya-u.ac.jp/MICAN.

## Competing interests

The authors declare that they have no competing interests.

## Authors’ contributions

SM designed and carried out research and drafted manuscript. KS discussed and helped to design MICAN methodology. GC coordinated and designed research and drafted manuscript. All authors read and approved the final manuscript.
